# Estimating the number of principal components via Split-Half Eigenvector Matching (SHEM)

**DOI:** 10.1016/j.mex.2023.102286

**Published:** 2023-07-08

**Authors:** Thomas E. Gladwin

**Affiliations:** Experience Design Team, Institute for Globally Distributed Open Research and Education (IGDORE), Sopra Steria, 6th Floor, 1 Bartholomew Close, EC1A 7BL London, United Kingdom

**Keywords:** Principal component analysis, Dimension reduction, Number of components, Eigenvectors, SHEM: Split-Half Eigenvector Matching

## Abstract

Estimating the number of principal components to retain for dimension reduction is a critical step in many applications of principal component analysis. Common methods may not be optimal, however. The current paper presents an alternative procedure that aims to recover the true number of principal components, in the sense of the number of independent vectors involved in the generation of the data.

• Data are split into random halves repeatedly.
• For each split, the eigenvectors in one half are compared to those in the other.
• The split between high and low similarities is used to estimate the number of principal components.

The method is a proof of principle that similarity over split-halves of the data may provide a useful approach to estimating the number of components in dimension reduction, or of similar dimensions in other models.

## Graphical abstract

**Figure:**
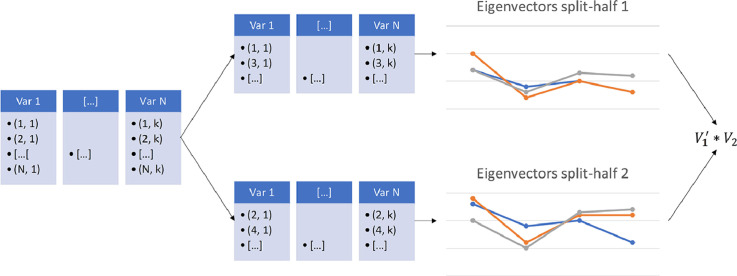

Specifications table

**Table:** 

Subject area:	Mathematics and Statistics
More specific subject area:	Principal Component Analysis
Name of your method:	SHEM: Split-Half Eigenvector Matching
Name and reference of original method:	Not applicable.
Resource availability:	https://github.com/thomasgladwin/teg_get_best_n

## Method details

Principal Component Analysis (PCA) is a commonly used method for dimension reduction, using only basic vector algebraic methods. A fundamental question when using PCA for dimension reduction is how many components should be retained. A common heuristic uses the shape of the curve of ordered eigenvalues, referred to as scree or elbow tests. This is simple and convenient, but concerns have been raised about the accuracy of the procedure and implications of incorrect values, e.g., in the context of evolutionary biology research [1]. Improvements to scree plots have been suggested [5,8] and methods have been proposed that use permutation or randomization tests to estimate and test the number of components [2,7,9]. Going beyond PCA, factor analysis provides statistical model-testing procedures that could be used to decide on the number of latent variables [3,4].

An, in a sense intermediate, method is presented here, that aims to be more principled and accurate than eigenvalue-based criteria but still within the PCA rather than factor analysis approach. The method uses comparisons between the dataset's split-half eigenvectors. The rationale is that the same true latent variables should tend to be found in random splits of the data. The aim is to serve as a proof of principle of this alternative type of approach to the problem.

The procedure is conceptually simple, and consists of the following steps; Python code is available on GitHub [6].

- The data matrix, of size (number_of_observations x number_of_variables), is split into random halves of subsets of observations. This is repeated a given number of times.
- For each split, principal component analysis is applied to both halves of the data.
- For each eigenvector in the first half of the data, the highest inner product is found with an eigenvector in the second half of the data. This provides a measure of split-half similarity for each principal component. Eigenvectors may only be matched once.
- The mean of the similarity-vectors over all random splits is taken.
- The mean similarity vector is divided into high- versus low-similarity sections. This is done by calculating a separation score for each index of the mean similarity vector. The score is B / W, where B is the “between” variance and W is the “within” variance. B is the variance of the vector in which each element is the mean value of the high section (elements up to, but not including, the current index) or the low section (the current index to the end of the vector). W is the sum of the variance within each section.
- The number of components is defined to be the separating index with the highest separation score.
- An additional heuristic step aims to identify the case of zero true latent components. To do so, a second adjusted separation score is created for each index, which scales the score by 1 – (index – 1)/ number_of_variables. If the adjusted separation score associated with the index with the highest separation score is below 1, then the number of components is set to 0. This step follows the rationale that a high number of components will be theoretically unlikely as well as low in parsimony.

The method was validated using simulated data. Simulated data were generated as a (number_of_observations x number_of_variables) matrix of independent normally distributed data, with standard deviation SD_noise, to which a specified number of “true”, generating latent variables were added. Each latent variable was a standard normal random column vector, added to the data after being multiplied by a row vector of standard normal random coefficients. 100 simulations were run, for 1000 observations, 100 variables, and SD_noise = 0.5. Results are shown in Table 1 below.

**Table 1:** Method validation results.

True number	Mean estimate	Proportion zero-case	Accuracy (non-zero)
0	44.33	1	0
1	1	0	1
2	2	0	1
3	3	0	1
4	3.99	0	0.99
5	4.92	0	0.96
6	6	0	1
7	6.97	0	0.97
8	7.98	0	0.98
9	8.99	0	0.99
10	9.97	0	0.97
15	14.97	0	0.97
20	19.73	0	0.73

*Note*. Each row of the Table shows the results for a given true number of latent components used to generate the data. “Mean estimate” is the mean, over simulations, of the estimated number of components. “Proportion zero-case” is the proportion of simulations in which the procedure flagged that there were zero generating components; this indeed occurred in the case when there were no generating components. “Accuracy” is the proportion of simulations in which the estimated number of components (not considering zero-case flags) was the same as the true number of components.

Thus, the SHEM procedure can recover the true, i.e., generating, number of latent variables for the simulated situation, although it seems to work best when the true number is relatively low. The situation with zero generating components was flagged by the heuristic for zero-cases. Accuracy in practice will depend on the combination of the number of observations, the number of variables, the true number of dimensions, and noise. Adjusted simulations could be run to assess the applicability of the method for a given design.

A judgment is required when a zero-case is flagged – there will in general be uncertainty whether there are no generating latent components or relatively many true components. Which kind of error poses the greater risk, or what number of components would be theoretically plausible or useful in terms of parsimony, could lead to the decision to use the zero-estimate or not. For instance, it may be a priori implausible for there not to be at least one underlying latent variable causing correlations between observed variables, or the data require dimension reduction to a certain maximum acceptable number of dimensions to be useful for subsequent analyses.

In conclusion, using similarity over randomly split data may be a useful approach to estimate the true number of components. Using split-similarity, rather than using heuristics involving patterns found for the whole dataset, may be generalizable to similar problems involving the need to select parameters, such as the number of clusters in k-means clustering.

## Ethics statements

Not applicable.

## CRediT author statement

Thomas Gladwin: Conceptualization, Methodology, Software, Writing.

## Declaration of Competing Interest

The authors declare that they have no known competing financial interests or personal relationships that could have appeared to influence the work reported in this paper.

## Data Availability

No data was used for the research described in the article.
